# How do high-performance work systems affect work fatigue: The mediating effect of job responsibility and role overload

**DOI:** 10.1371/journal.pone.0269452

**Published:** 2022-07-06

**Authors:** Sun Jiandong, Xuexiu Fan, Li Haitian

**Affiliations:** 1 School of Management, Guilin University of Aerospace Technology, Guilin City, Guangxi Zhuang Autonomous Region, China; 2 School of Foreign Language and International Business, Guilin University of Aerospace Technology, Guilin City, Guangxi Zhuang Autonomous Region, China; 3 School of Pharmacy, Southwest Medical University, Luzhou, China; University of Education, PAKISTAN

## Abstract

Work fatigue refers to physical, mental, and emotional exhaustion, resulting in the inability to work. Hitherto research indicate a close relationship between high-performance work systems and work fatigue, and there may be a double-edged sword effect of high-performance work systems on work fatigue. However, a comprehensive theoretical framework has not been developed to understand the relationship between them. Based on the challenge-hindrance stress model, this study employs role overload and job responsibility as mediating variables in a conceptual framework to understand the impact of high-performance work systems on work fatigue. Using the partial least square structural equation model and a sample of 360 employees in China, the mediating effects of role overload and job responsibility were confirmed. Further, the internal mechanisms of how high-performance work systems affect work fatigue are discussed, its adverse effects are confirmed, and its practical implications are proposed.

## Introduction

Work fatigue refers to the physical, mental, and emotional exhaustion caused by stressors at work, resulting in the inability to highly effective work [1]. Reducing and managing employees’ work fatigue through effective management practices remains an important goal pursued by both enterprises and scholars, because work fatigue is detrimental to employees’ physical health [2, 3], work attitude, work safety, and work performance [4]. Although numerous factors influence employees’ physical and psychological statuses, such as their leaders and organizational climates, human resource policies and practices are easier to control. Human resource management, especially high-performance work systems (HPWS), which are regarded as the best human resource management practices, are considered effective in ensuring employee welfare [5]. Therefore, since its emergence, not only have scholars applauded HPWS highly, but a large number of enterprises have also implemented it to ensure their sustainability and development.

In recent years, with the increasing number of occupational diseases and frequent occurrence of management events, for instance, the "sudden death of programmers in China," some scholars began to rethink the relationship between HPWS and work fatigue, and two different views emerged. One perspective is that HPWS provides employees with work autonomy and opportunities for work participation that are attached to their job responsibilities, which give them a sense of achievement after completing their work, thus reducing fatigue [6]. Another perspective is that HPWS requires employees to complete diversified work within a limited period, which causes them to perceive a role overload and quickly depletes their energy, thus causing more fatigue [7–9]. However, although hitherto research indicated a double sword effect of HPWS on work fatigue, there is no appropriate theoretical framework helping us understand the relationship between HPWS and work fatigue.

Work stress has long been recognized to relate closely to work fatigue. Moreover, according to challenge-hindrance stress model, challenge stress, such as job responsibility, often causes positive organizational results, while hindrance stress, such as role overload, causes adverse organizational outcomes [10]. Therefore, this study leverage challenge-hindrance stress model and employs job responsibility and role overload as mediating variables in a theoretical framework to understand the double- sword effect of HPWS on worker fatigue. Through this paper, we contribute to the research on HPWS as well as work fatigue and offer some guidance for enterprises on its effective implementation.

## Theoretical background and hypotheses formulation

### HPWS and work fatigue

HPWS refers to a bundle of human resource practices aiming at improving employees’ performance, including strict recruitment and selection processes, extensive training, employee participation and authorization, performance-based evaluation, and information sharing [11]. Initial studies have suggested that HPWS not only improves enterprises’ performance but also has a positive impact on employees’ well-being; therefore, it is a win-win management model [12]. In recent years, the negative outcomes of HPWS, such as work intensification, emotional exhaustion, have gradually been unearthed [13, 14]. Although there is no direct evidence proving that HPWS has a double-sword effect on work fatigue, there are still some evidences indicate such trend.

A stream of research indicate that HPWS can alleviate the work fatigue. Some research found that HPWS can endow employees with work autonomy, which can help them modify their work schedule and work model [14, 15], eventually reducing the fatigue caused by intensive work [16]. Other research revealed that the information of organizational norm translated by HPWS will trigger the procedural justice perceived by employees [17], which is a vital precursor of low work fatigue [18].

Another stream of research, in contrast, criticized that HPWS reinforces work fatigue in the workplace. Ramsay, Scholarios [9] held the view that HPWS intensifies the work of employees and causes burnout, triggering a increased work fatigue subsequently. Based on the job demand-control model, Jensen, Patel [8] found that in a circumstance of HPWS, employees are more likely to generate a perception of role overload. Van De Voorde and Beijer [14] leveraged attribution theory to confirm that HPWS have a adverse influence on the physical and mental health of employees.

### Challenge—hindrance stress model

Stress refers to the extent to which individuals respond to emotional or physical stimuli from their external environment. Work-related stress usually refers to employees’ direct response to external stressors, such as work requirements, practice requirements, and performance appraisals [19]. Early studies on stress mainly focused on the negative consequences of work stress, including turnover and job dissatisfaction; however, results from empirical studies indicate that the correlation between stress and these negative consequences may not always be significantly positive [20, 21]. As such, researchers have considered whether all types of stress lead to negative consequences. For example, Selye [22] divided stress into "positive stress" and "negative stress" and proposed that positive stress would cause positive organizational consequences while negative stress would cause negative outcomes. Lazarus and Folkman [23] argued that individuals evaluate their current stressful situations and classify them as either challenging situations or hindrance situations.

Based on previous studies, Cavanaugh, Boswell [24] proposed the concepts of challenge and hindrance stress. Challenge stress refers to the stress that is within employees’ control, such as job responsibility and time pressure, which can help employees set goals and encourage them to learn. Employees can take full advantage of their potential and progress by overcoming this stress [24, 25]. However, hindrance stress refers to the stress that employees are unable to overcome because of their inability to handle it, which mainly includes role overload, role conflict, and workplace contradictions. Hindrance stress impedes employees from accomplishing work tasks and is detrimental to their job satisfaction and health [26]. Prior studies have confirmed that employees who face challenge stress report higher job performance, job satisfaction, job engagement, and a lower turnover intention than those who encounter hindrance stress [27, 28].

### The mediating effect of role overload

Role overload refers to the degree to which employees face role expectations, in their jobs, that they cannot achieve [29]. On perceiving role overload, employees invest more time and energy to meet these external expectations, which causes a rapid depletion in their physical and mental energy and, subsequently, fatigue [30]. Further, being in a state of prolonged hindrance stress reduces employees’ focus and increases their possibility of making mistakes [31]. Under the enterprise’s existing performance evaluation system, employees perceive higher work stress and they become trapped in a vicious spiral. As a result, they may become more fatigued. Moreover, due to long-term role overload, employees assign more time and energy to work and reduce the time and energy allocated to family life, which may cause family conflicts, reduce the quality of rest, and exacerbate their sense of fatigue [32].

The employees’ role overload is mainly affected by two factors: the inability of employees to meet their work requirements and the time pressure they encounter [33]. In enterprises, the HPWS emphasizes allocating more work directions and diversity to employees, which somewhat complicates the employees’ work requirements [34]. Employees must have diversified working abilities to confront various work requirements and complete their work, which leads to the perception that their skill levels or knowledge scope cannot effectively meet the work requirements proposed by HPWS. In addition, the flexible working time emphasized by HPWS not only provides employees with the freedom to choose their working time but also makes it more difficult for employees to organize their time [35]. Moreover, employees usually take up tremendous work due to the profit-seeking orientation followed by enterprises and thus are unable to complete the work within the limited working time, which exacerbates their perception of time pressure [35]. In other words, HPWS not only intensifies the demands on employees’ ability but also aggravates their time pressure, and as such, employees in an enterprise that adopts HPWS may feel more role overload. Therefore, we propose the following hypotheses:

H1: HPWS is positively associated with role overload, which acts as a mediator for more work fatigue.

### The mediating role of job responsibility

Job responsibility refers to the extent to which employees assume responsibility for their work results [36]. As a type of challenge stress, job responsibility helps employees to generate more positive feelings and relieve their work fatigue. First, faced with work requirements, employees evaluate their ability to cope with the requirements and the potential returns after meeting them. If they are able to meet the requirements using their efforts and gain more returns, such as improved working skills and increased salaries, they feel a sense of achievement and become motivated [37]. Therefore, when employees undertake job responsibilities, they are willing to invest their energy and gain a greater sense of achievement, as they believe that they can adapt to the pressure, through their efforts, and receive rewards, such as skill improvement, salary increments, and promotions, which can effectively reduce their fatigue [37]. In addition, in an enterprise, employees often want to establish good relationships with colleagues and supervisors and obtain their approval. Fulfilling job responsibilities well is an essential approach to obtaining approval and meeting emotional needs [38]. From this perspective, job responsibility can also stimulate employees’ intrinsic motivation, making them feel more work satisfaction, thus reducing fatigue.

In enterprises, HPWS, as an indispensable component of the workplace, is usually an important source from which employees perceive job responsibilities. For example, Boxall and Macky [39] believed that in the HPWS, work participation requires employees to make choices regarding their work, which means that they should be responsible for their work results. As such, employees perceive that their responsibilities increase. Moreover, including comprehensive training, performance rewards, participation in decision-making, information sharing, and teamwork in the HPWS can make employees feel more authoritative [40, 41], which may increase their perceived job responsibilities. Based on the above analysis, this study proposes the following hypotheses:

H2: HPWS will be positively associated with job responsibility, which acts as a mediator of less work fatigue.

## Method

### Sample and procedures

We collected survey data from enterprises in China through paper-and-pencil surveys during June 2019 to March 2020 and February 2021 to March 2021. First, using e-mails or phone calls, we requested some familiar human resource managers to help us recruit some voluntary participators in their enterprises. Eventually, 14 human resource managers helped us recruit participants and organize the survey. Subsequently, we visited the enterprises to conduct the survey. During the process of survey, simple random sampling technique was used to select samples. Specifically, we code every employee who are willing to participate in the research, and then use the random number table to select participants. To ensure survey quality, we promised participants at the beginning of the survey that the data would only be used for academic research and assured them that the questionnaires did not contain any identifying information. The administrative staff was also requested to leave when participants were filling in the questionnaires. Overall, we collected 400 questionnaires. The data were then cleaned in two ways: lie detection items were set in the questionnaires, and questionnaires that contained more than six consecutive identical answers were deleted. Finally, 40 invalid questionnaires were excluded, and 360 valid questionnaires were retained. The basic information in the valid samples was as follows: from the gender perspective, males account for 61.1% and females account for 38.9% of the total. As for age, 18–30 years old account for 71.9%, 31–40 years old account for 22.8%, 41–50 years old account for 2.8%, and 51–60 years old account for 2.5%. As for tenure years, 73.3% had under 5 years, 20.8% had 6–10 years, and 5.8% had more than 11 years. As for education level, 6.9% were high school graduates and below, 23.6% had associate degrees, 61.4% were undergraduates, and 8.1% were postgraduates and above.

### Ethics approval

An ethics approval was not required according to institutional guidelines and national laws regulations. First, we just conducted questionnaire surveys, and our research did not involve human clinical trials or animal experiments. Second, the content of the questionnaire did not involve any personal privacy, ethical and moral topics. Third, we informed the participants about the objectives of the study and guaranteed their confidentiality and anonymity. Fourth, All the participants were completely free to join or drop out of the survey. Only those who were willing to participate were recruited.

### Measures

In this study, all variables are measured using popular scales frequently used worldwide. Among them, the English scale has been revised to Chinese using translation and back-translation procedures. All scales were measured using a 5-point Likert scale, with 1 point representing complete disagreement and 5 points representing complete agreement with the description of the question.

1. High-performance Work System (HPWS): The HPWS adopts the scale designed for Chinese employees by Sun, Aryee [11], which contains 24 items. Sampled items include "The company provides comprehensive training for employees" and "the company pays employees according to their performance appraisal results." The Cronbach’s coefficient for this scale was 0.973.
2. Role Overload: Role overload was measured using the scale translated from Peterson, Smith [41] by LI and Zhang [42]. The scale consists of five items, including "It is necessary to relieve part of my work" and "My work burden is too heavy." The scale has been proven to have good reliability and validity in previous studies. The Cronbach’s coefficient for this scale was 0.908.
3. Job Responsibility: We added one item to the scale used by Oppenauer and Van De Voorde [37] as well as Cavanaugh, Boswell [24] to form the job responsibility scale, which consists of three items. These items include "I work in the considerable responsibility," "If the work at hand is not successful, I will have considerable responsibility," and "My responsibilities range is very wide. " The scale includes both the depth and breadth of the job responsibilities. The Cronbach’s coefficient for this scale was 0.915.
4. Work fatigue: The work fatigue measurement adopts the three-dimensional work fatigue inventory scale designed by Frone and Tidwell [1] which contains 18 items. The scale has been tested under different cultural backgrounds showing good reliability [43]. The main sample items include "I do not want to do anything after work, including important things" and "I feel psychologically tired after work." The Cronbach’s coefficient for this scale in this study was 0.984.
5. Control Variables: In this study, demographic characteristics, such as gender, age, working years, and education level, were selected as control variables to exclude the influence of other factors on work fatigue.

### Data analysis

#### Common method biases test

During the investigation, we explained the purpose of the test in detail to the employees and set two lie test items to screen out invalid questionnaires. However, because the data used were all filled at the same time point, common method biases may still exist. In this study, Harman one-way analysis of variance [44] was used to test the common method bias, and the explained variance of the first factor without rotation was 38.206%, which is less than 40%. As observed, the common method biases in the data are not significant, and further statistical analysis can be conducted.

#### Discriminant validity of variables

To test the good discriminative validity among variables, Mplus 8.3 was used to conduct confirmatory factor analysis on HPWS, job responsibility, role overload, and work fatigue. The results of the confirmatory factor analysis are presented in Table 1. As shown, the fitting degree of the four-factor model is the best, indicating that the four variables have good discriminant validity.

**Table 1:** Comparison of confirmatory factor analysis.

model		*χ* ^2^	df	χ^2^/df	RMESA	CFI	TLI	SRMR
1	Four-factor model(F1,F2,F3,F4)	2877.718	1169	2.462	0.064	0.915	0.911	0.043
2	Three-factor model(F1+F2,F3,F4)	4031.696	1172	3.440	0.082	0.859	0.852	0.075
3	Three-factor model(F2, F1+F3,F4)	3640.344	1172	3.106	0.076	0.878	0.872	0.062
4	Three-factor model(F1,F2+F3,F4)	3661.549	1172	3.124	0.077	0.877	0.871	0.073
5	Two-factor model (F1+ F3+F2,F4)	4751.362	1174	4.047	0.092	0.823	0.815	0.084
6	Two-factor model (F1,F3+ F2+F4)	5067.955	1174	4.317	0.096	0.807	0.799	0.132
7	Single factor model (F1+F2+F3+F4)	13130.889	1175	11.175	0.168	0.409	0.383	0.253

Note: F1 refers to HPWS, F2 refers to role overload, F3 refers to job responsibility, F4 refers to work fatigue, and "+" refers to the combination of two variables.

## Results

The mean values, standard deviations, and correlation coefficients of the variables used in this study are shown in Table 2, which reveals that HPWS is positively correlated with role overload (r = 0.370, p<0.05) and job responsibility (r = 0.440, p<0.05). It was also found that role overload is positively correlated with work fatigue (r = 0.417, p<0.05). The logic of H1 is preliminarily reflected. Job responsibility is not correlated to work fatigue (r = -0.051, p<0.05), and whether HPWS influences work fatigue through job responsibility requires further exploration.

**Table 2:** Mean value, standard deviation, and correlation coefficient.

	Mean	SD	1	2	3
1.HPWS	3.60	0.50			
2.Role overload	3.49	0.62	0.370**		
3.Job responsibilities	3.48	0.75	0.440**	0.417**	
4.Work fatigue	3.11	0.74	0.236**	0.258**	-0.051

**Fig 1:**
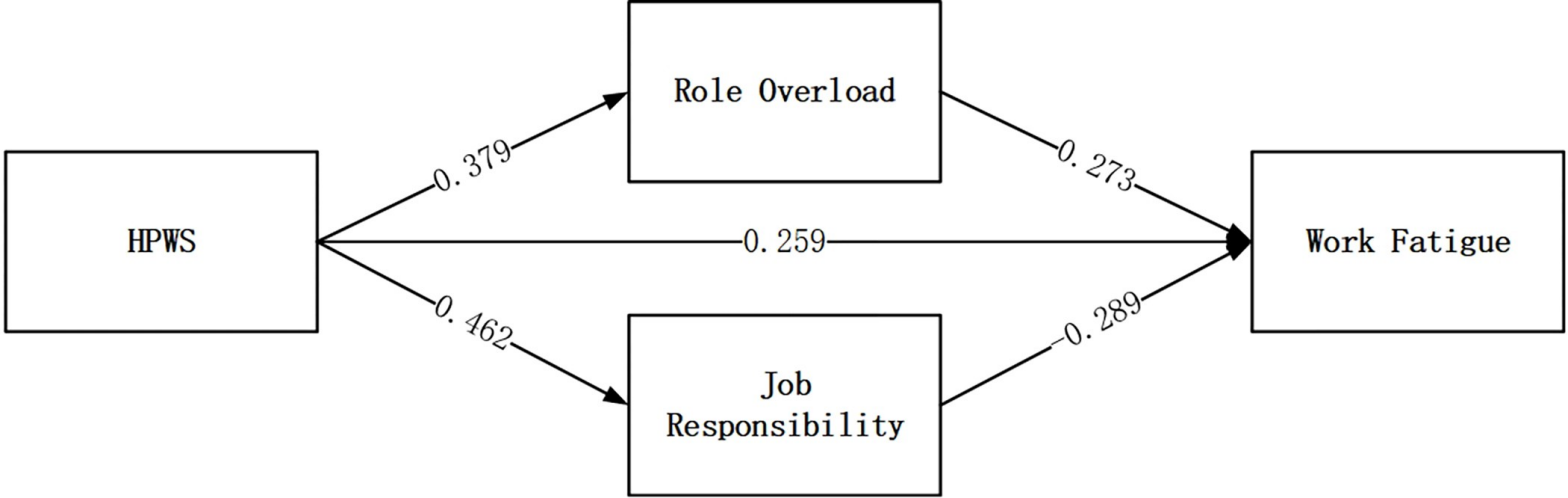


In this study, Mplus 8.3 is used for path analysis of data, and the path analysis result is shown in Fig 1. H1 proposed that HPWS has a positive effect on work fatigue through role overload. As shown in Fig 1, HPWS has a positive influence on role overload (beta = 0.379, P <0.05), which means that role overload continuously increases with the HPWS increase. Further, role overload has a positive effect on work fatigue (beta = 0.273, P <0.05), indicating that work fatigue increases with the increase in role overload. The mediating effect analysis (Table 3) shows that the mediating effect value of role overload between HPWS and work fatigue is 0.104, with a 95% confidence interval of (0.052, 0.162). Therefore, the mediating effect of role overload is valid, supporting H1.

**Table 3:** Mediating effect test of role overload and job responsibility.

path	effect	SE	P	Confidence interval (95%)	
				BootLLCI	BootULCI
HPWSs→ Role overload → Work fatigue	0.104	0.028	0.000	0.052	0.162
HPWSs→ Job responsibility → Work fatigue	-0.133	0.031	0.000	-0.195	-0.073
HPWSs →Work fatigue	0.259	0.061	0.000	0.137	0.381

According to H2, HPWS alleviates work fatigue by enhancing job responsibilities. As shown in Fig 1, HPWS has a positive influence on job responsibility (beta = 0.462, P <0.05), indicating that job responsibility is constantly increasing with HPWS. Job responsibility has a negative effect on work fatigue (beta = -0.289, P <0.05), indicating that work fatigue gradually decreases with job responsibility. The mediating effect of job responsibility between HPWS and work fatigue is -0.133, and the 95% confidence interval is (-0.195, -0.073). Therefore, the mediating effect of job responsibility is statistically significant, and H2 is supported.

## Discussion

Based on the challenge-hindrance stress model, this paper discusses the double-edged sword effect of HPWS on work fatigue and verifies the mediating roles of role overload and job responsibility. The empirical results indicate that employees perceive higher role overload in enterprises that adopt HPWS, due to more ability and time requirements, which causes them to exert more time and energy into their work and consequently causes fatigue. On the other hand, the discretions and participation emphasized by HPWS will cause employees to perceive a sense of responsibility, further stimulating their intrinsic motivation and meeting their emotional needs. As a result, employees’ sense of fatigue will be effectively reconciled. The results of this study contribute to the understanding of how HPWS influences work fatigue and reveal its negative side.

### Theoretical implication

First, this study contributes to the literature on the double-edged sword effect of HPWS by employing job responsibility and role overload as its consequence variables. With the enrichment of research on HPWS, two main perspectives emerged. The "win-win perspective" holds that HPWS can improve enterprise performance and nourish employees’ well-being simultaneously, while the "criticism perspective" suggests that HPWS increases employees’ work intensity and strengthens exploitation on them [12]. In recent years, some scholars aggregated these two views and believed that the impact of HPWS on employees’ feelings and well-being is a double-edged sword effect based on the two perspectives mentioned above [34], but a lack of empirical research undermines the validity of such view. The results in this study indicate that HPWS has two contradictory impacts on work fatigue: it accentuates work fatigue by increasing the role overload perceived by employees, and it attenuates work fatigue by increasing job responsibility, supporting the view of double-edged sword effect. The results of this study also echo the research by Topcic, Baum [45], who argued that while the discretion and employee participation emphasized by HPWS has a positive impact on employees, it also has a negative impact on employees’ health by increasing work demands and time pressure. This study supplements the current research, which considers the double-edged sword effect of HPWS and offers a new direction for future research on its influence on employees’ attitudes and behaviors.

Second, challenge-hindrance model was been introduced to understand how HPWS influence employees’ attitudes and behaviors. In recent years, work stress has been considered an important mechanism through which HPWS negatively affects employee health and well-being [37]. However, different types of stress may have different impacts on employees in the workplace [46]. Simply identifying stress as a negative outcome caused by HPWS is not conducive to further understanding its working mechanism and improvement. Based on the hindrance-challenge stress model, this study selects role overload and job responsibility as mediating variables and proves the double-edged sword effect of HPWS on work fatigue. This paradigm involving two types of stress not only provide a new insight to understand the relationship between HPWS and work fatigue, but also offer a new approach for future research to explore how HPWS influence employees’ job attitude and behaviors.

### Practical implication

There are three main managerial suggestions for organizations that have adopted HPWS.

First, according to this study’s results, the impact of HPWS on employee fatigue is a double-edged sword effect, that is, HPWS increases work fatigue through role overload but also reduces work fatigue through job responsibility, simultaneously. Therefore, enterprises should focus on the HPWS practice process. In addition, to ensure that employees experience work autonomy and work participation, enterprises should clarify employees’ work content and work objectives to ensure that the requirements they confront are within their scope. As such, the hindrance stress perceived by employees can reduce, and the challenge stress perceived by employees increase. Consequently, employees feel less fatigued, which could ensure their efficiency and health and help the enterprise maintain sustainable growth.

Second, prior studies also show that social support within an enterprise, including support from leaders, colleagues, or unions, can effectively reconcile the negative effects caused by HPWS [12]. When employees receive more support in their workplace, they tend to regard work requirements as challenge stress, which can motivate them to work efficiently and reduce fatigue [37]. Therefore, enterprises should be aware of the role of leaders and thus pay more attention to training grass-root leaders, ensuring that they can provide technical and resource support to employees when necessary [47]. In addition, enterprises should also strengthen contacts between departments, organize more communication activities, and help employees establish better interpersonal relationship-networks in their enterprises. When employees assist each other, they complete their work tasks efficiently and feel less work fatigue [48].

Third, enterprises should recognize that every managerial practice possesses potential negative effect and carefully control it. Today, many enterprises are fascinated by various human resources management bundle, such as high-performance work systems, high-involvement work systems, and believe the totipotency of these bundles of management practices. However, the results of this study, as well as results of many previous research, indicate that there are no perfect management systems [8, 49]. Only a proper utilization of practices will ensure the effectiveness of enterprises and well-being of employees. Therefore, it is a propriety of using suitable management practices, but not solely pursuing fashionable management systems, that can help enterprises survive, develop, and succeed in such a capricious environment.

#### Limitations and directions for future research

This study has some limitations. First, the cross-sectional study design limits the validity of the causal relationships between variables. The four variables involved in this study, HPWS, role overload, job responsibility, and work fatigue were all measured at only one point in time. Although this method somewhat reflects the relationship between variables, it cannot fully reflect the causal relationships between variables. Moreover, the influence of stress tends to have persistent and lagging effects on employees [30]. Therefore, longitudinal multi-time point tracking studies can be adopted in subsequent studies to understand the impact of HPWS on work fatigue through stress more accurately. Second, during the hypotheses test, we found that HPWS has a direct positive effect on work fatigue, in addition to positive effects through role overload and negative effects through job responsibility. As noted, using other mediating variables, we can understand how HPWS fully influences work fatigue. Future studies could explore the mediating role of other stress-related variables between HPWS and work fatigue. Finally, this study only analyzes the internal mechanism of HPWS on work fatigue; it does not examine the boundary conditions of this mechanism. To develop a theoretical framework for the impact of HPWS on work fatigue, future studies should explore the boundary condition of the relationship between them, such as the type of enterprise, leaders, and other contextual components.

## Conclusion

Hitherto research implicit a double sword effect of HPWS on work fatigue, but there is no empirical study construct a framework to understand the mechanism of the relationship between them. This study leveraged challenge-hindrance stress model and integrate job responsibility and role overload as mediating variables in the framework to help understand how HPWS influence work fatigue. The results of this study confirm the framework proposed by this study. Future studies should expand and enrich the research on the relationship between HPWS and work fatigue by leveraging other theories, such as attribution theory or JD-R model.

## Supporting information

S1 Data (XLSX)
